# Multi‐omics profiling of Alzheimer’s disease in multi‐ethnic populations

**DOI:** 10.1002/alz.090590

**Published:** 2025-01-03

**Authors:** Joseph S. Reddy, Laura M Heath, Mariet Allen, William L. Poehlman, Yanling Wang, Ryan Johnson, Erming Wang, Yiyi Ma, Charlotte C.G. Ho, Katia de Paiva Lopes, Abby Vander Linden, Zachary Quicksall, Xianfeng Chen, Saurabh Baheti, Thuy Nguyen, Geovanna Yepez‐Sisalema, Adriana O Mitchell, Stephanie R Oatman, Xue Wang, Minerva M. Carrasquillo, Alexi Runnels, Andrew F Teich, Thomas G. Beach, Geidy E Serrano, Todd Golde, Lisa L. Barnes, Minghui Wang, Vahram Haroutunian, Bin Zhang, Philip L. De Jager, David A. Bennett, Nicholas T Seyfried, Anna Greenwood, Nilüfer Ertekin‐Taner, Accelerating Medicines Partnership for Alzheimer’s Disease (AMP‐AD) Diversity Initiative

**Affiliations:** ^1^ Mayo Clinic, Jacksonville, FL USA; ^2^ Sage Bionetworks, Seattle, WA USA; ^3^ Rush Alzheimer’s Disease Center, Rush University Medical Center, Chicago, IL USA; ^4^ Rush University, Chicago, IL USA; ^5^ Icahn School of Medicine at Mount Sinai, New York, NY USA; ^6^ Center for Translational & Computational Neuroimmunology, Department of Neurology, Columbia University Medical Center, New York City, NY USA; ^7^ Mayo Clinic, Phoenix, AZ USA; ^8^ Mayo Clinic, Rochester, MN USA; ^9^ New York Genome Center, New York, NY USA; ^10^ Columbia University, New York, NY USA; ^11^ Banner Sun Health Research Institute, Sun City, AZ USA; ^12^ Civin Laboratory for Neuropathology, Banner Sun Health Research Institute, 10515 W Santa Fe Drive, Sun City, AZ 85351, Sun City, AZ USA; ^13^ Emory University, Atlanta, GA USA; ^14^ Rush Alzheimer’s Disease Center, Chicago, IL USA; ^15^ Center for Translational & Computational Neuroimmunology, Columbia University Irving Medical Center, New York, NY USA; ^16^ Emory University School of Medicine, Atlanta, GA USA

## Abstract

**Background:**

Alzheimer’s disease (AD) is a devastating neurodegenerative disorder with few therapies to treat, mitigate or prevent its onset. Understanding of this disease is predominantly based on research in non‐Hispanic Whites (NHW) although AD disproportionately affects African Americans (AA) and Latin Americans (LA), underrepresented in AD research. To address this knowledge gap, the Accelerating Medicine Partnership for Alzheimer’s Disease (AMP‐AD) Diversity Working Group was launched to generate multi‐omics data from post‐mortem brain tissue from donors of predominantly AA and LA descent. Here, we focus on genomic and transcriptomics measures, made available through the AD Knowledge Portal (ADKP).

**Method:**

Brain tissue from dorsolateral prefrontal cortex (DLPFC), caudate nucleus (CN), superior temporal gyrus (STG) and temporal pole (TP) regions of AA (N = 299), LA (N = 344) and NHW (N = 532) donors was obtained through Mayo Clinic, Mt. Sinai School of Medicine, Emory, Columbia, Rush Alzheimer’s Disease Center, Banner Sun Health, and 1Florida. DNA extracted from DLPFC tissue samples (n = 641) was utilized for whole genome sequencing (WGS) at New York Genome Center (NYGC). RNAseq of 2,224 samples was conducted either at Mayo, Rush or NYGC. Sequencing reads were processed through GenomeGPS and MAPRseq pipelines for WGS and RNAseq data, respectively. Following quality control (QC), RNAseq data was normalized and assessed for sources of variation (SOV). Integration of WGS and RNAseq data to perform comparative eQTL analysis across multi‐ethnic populations and brain regions is ongoing.

**Result:**

WGS data for 621 donors, and RNAseq data generated at Mayo (n = 1,136 samples), Rush (n = 627), and NYGC (n = 779) sequencing centers, were deposited to the ADKP. Consensus processing of RNAseq data revealed site, sex, race/ethnicity, RIN, AD diagnosis, age at death and/or library batch as SOV across various sites and brain regions. Principal component analysis of gene expression measures revealed no major stratification by disease or brain region. Accompanying clinical metadata of donors have been distributed through ADKP.

**Conclusion:**

These multi‐omics data made available to the research community is expected to be an initial step towards bridging our data and knowledge gap to elucidate conserved and distinct AD related pathways across these underrepresented at‐risk populations.

